# The Amyloid as a Ribbon-Like Micelle in Contrast to Spherical Micelles Represented by Globular Proteins

**DOI:** 10.3390/molecules24234395

**Published:** 2019-12-03

**Authors:** Mateusz Banach, Leszek Konieczny, Irena Roterman

**Affiliations:** 1 Department of Bioinformatics and Telemedicine, Jagiellonian University-Medical College, Lazarza 16, 31-530 Krakow, Poland; mateusz.banach@uj.edu.pl; 2 Chair of Medical Biochemistry, Jagiellonian University-Medical College, Kopernika 7, 31-034 Krakow, Poland; mbkoniec@cyf-kr.edu.pl

**Keywords:** amyloid, fibril, tau, synuclein, hydrophobicity, hydrophobic core, spherical micelle, ribbon-like micelle, symmetry

## Abstract

Selected amyloid structures available in the Protein Data Bank have been subjected to a comparative analysis. Classification is based on the distribution of hydrophobicity in amyloids that differ with respect to sequence, chain length, the distribution of beta folds, protofibril structure, and the arrangement of protofibrils in each superfibril. The study set includes the following amyloids: Aβ (1–42), which is listed as Aβ (15–40) and carries the D23N mutation, and Aβ (11–42) and Aβ (1–40), both of which carry the E22Δ mutation, tau amyloid, and α-synuclein. Based on the fuzzy oil drop model (FOD), we determined that, despite their conformational diversity, all presented amyloids adopt a similar structural pattern that can be described as a ribbon-like micelle. The same model, when applied to globular proteins, results in structures referred to as “globular micelles,” emerging as a result of interactions between the proteins’ constituent residues and the aqueous solvent. Due to their composition, amyloids are unable to attain entropically favorable globular forms and instead attempt to limit contact between hydrophobic residues and water by producing elongated structures. Such structures typically contain quasi hydrophobic cores that stretch along the fibril’s long axis. Similar properties are commonly found in ribbon-like micelles, with alternating bands of high and low hydrophobicity emerging as the fibrils increase in length. Thus, while globular proteins are generally consistent with a 3D Gaussian distribution of hydrophobicity, the distribution instead conforms to a 2D Gaussian distribution in amyloid fibrils.

## 1. Introduction

The phenomenon of protein misfolding appears to be closely related to amyloid transformation, which is implicated in a number of neurodegenerative diseases [1]. Such conditions result from pathologically aggregated protein plaques referred to as amyloids [2]. A study of 50 different disorders and their symptoms revealed the propensity of certain proteins to produce amyloid plaques in standard environmental conditions [3]. Amyloid transformation is intimately tied to the process of in vivo protein folding [4,5,6]. The structural order of amyloids, where elongated structures are capable of unrestricted growth, results in the potentially dangerous accumulation of large macromolecules that interfere with biological mechanisms [7].

The high structural stability exhibited by amyloids is often attributed to the presence of numerous hydrogen bonds (the so-called cross-beta structures) [8]. The transformation of soluble proteins into fibrils can be explained as a gradual process where cross-beta forms emerge in a relatively disorganized environment and subsequently increase in size and breadth [9]. Intrinsically disordered proteins are therefore viewed as potential amyloid seeds. Indeed, such proteins may sometimes misfold, producing amyloid structures that appear more stable than their corresponding native forms [8,10].

According to the fuzzy oil drop model (FOD), upon which this publication is based, the structure of a globular protein may be likened to a spherical micelle, with a prominent hydrophobic core and a relatively hydrophilic surface. Such conditions are encountered, e.g., in antifreeze [11] and fast-folding proteins [12]. Biological function is often encoded as a localized deviation from the theoretical 3D Gaussian distribution of hydrophobicity (which models a “perfect” spherical micelle). Local hydrophobicity deficits often correspond to binding cavities that are capable of interacting with a ligand [13], while excess hydrophobicity—Especially if found on the protein surface—May indicate a potential complexation site [14,15].

The fuzzy oil drop model regards amyloid transformation as a shift between the monocentric core pattern and the linear distribution of hydrophobicity—And therefore between a spherical micelle and a ribbon-like micelle [16,17]. Theoretical speculations concerning changes in the parameters of the Gaussian distribution, discussed in [16], have been borne out by an analysis of a large variety of proteins (including DNA-binding proteins [17]) which detailed the methodology of applying the fuzzy oil drop model to protein structure analysis in general. The aforementioned work also revealed that results remained consistent regardless of the adopted intrinsic hydrophobicity scale [17]. While ribbon-like micelles also contain hydrophobic cores, the cores of these micelles specifically adopt the form of a linear hydrophobic band stretching along the fibril’s main axis. This was confirmed by the properties of fibrils listed in the Protein Data Bank (PDB) [18], each of which includes a hydrophobic band isolated from contact with the aqueous solvent.

A milestone in the history of protein folding research (before we move to misfolding) was the discussion introduced by Levinthal that formulated the idea of the so-called Levinthal’s paradox [19,20]. The analysis of micelle constructions seems to be an early step in research that is focused on the influence of water on polar molecules, such as amino acids [21]. The principles that govern the folding process of polypeptide chains, defined by Anfinsen, introduced fundamental rules for protein structuralization [22,23]. The importance of water, expressed by hydrophobicity, cast a new light on the folding process [24,25,26,27,28,29,30,31]. One consequence of this approach is the juxtaposition between the surface and the interior of proteins [32,33,34,35]. Levinthal’s paradox emerged as an important unsolved problem, especially in the era of computerized folding simulations where multicriteria optimization is a critical factor [36,37]. The role of the water environment is inexorably tied to the protein folding problem in general [38,39,40,41,42,43]. The idea of multi-step protein folding introduced the notion of intermediates in the folding process [44,45]. Despite progress in experimental and theoretical research, the opinion expressed by Chandler—“Despite the basic principles underlying the hydrophobic effect being qualitatively well understood, only recently have theoretical developments begun to explain and quantify many features of this ubiquitous phenomenon”—Seems to restrict the viability of qualitative solutions [46]. Both Levinthal’s paradox and the involvement of hydrophobic effects in protein folding have consistently been the focus of research in this field [47,48,49,50,51,52] ever since the misfolding phenomena were first recognized [53].

The discussion concerning protein folding in the context of water-to-protein relations would not be complete without mentioning the oil drop model defined by Kauzmann [54]. This model was later extended into the fuzzy oil drop model by introducing a 3D Gaussian function to express the hydrophobicity distribution in an idealized protein body [55].

The aim of this paper was the application of fuzzy oil drop model to the analysis of amyloid structures as listed in the PDB [18].

## 2. Results

### 2.1. FOD-Based Parameters to Interpret the Results

According to the fuzzy oil drop model, each protein molecule was characterized by several key parameters; particularly two variants of relative distance (RD): relative distance between observed (O) versus theoretical (T) and unified (R) (denoted as T–O–R) and observed (O) versus theoretical (T) and intrinsic hydrophobicity based distribution (H) (denoted as T–O–H). These parameters expressed the “distance” between the observed distribution of hydrophobicity (O) and a set of reference distributions, including the theoretical distribution (T)—The idealized one based on 3D Gaussian function, unified (R), and intrinsic hydrophobicity-based (H). For each residue, a value of T_i_ could be defined, a value which was the value of the Gaussian function at the position that corresponded to that molecule’s effective atom (averaged-out position of all atoms belonging to the residue). Since the “distance” between T and O was, in fact, a measure of entropy [56]. it could not be properly interpreted without another reference value. In our case, this value was provided by a uniform distribution denoted as R, which was, in a sense, “opposite” to T. Unlike the Gaussian function, R assigned an identical value of hydrophobicity to each residue. This yielded a value of RD for the T–O–R variant, telling us whether O more closely approximated the theoretical distribution (T) or the flat distribution (R). When RD < 0.5, we could assume that a prominent hydrophobic core was present within the protein molecule.

In a similar vein, RD (T–O–H) was derived by replacing the flat distribution (R) with a different type of reference distribution, where the value assigned to each residue was equal to its intrinsic hydrophobicity. Under these conditions, RD > 0.5 meant that the observed structure of the protein was dominated by the intrinsic properties of its constituent residues.

Other notable parameters determining the status of the molecule, as given by the fuzzy oil drop model, included a set of correlation coefficients expressing the relation between T and O (denoted as TvO), between H and O (denoted as HvO), and between H and T (denoted as HvT). Each coefficient told us to what degree the relevant distributions approximated each other.

When RD > 0.5 in both variants (T–O–R and T–O–H), we could say that the protein did not follow the Gaussian distribution and that its structural properties were determined by the intrinsic hydrophobicity of its component residues. This observation was usually reinforced by a high value of the HvO correlation coefficient.

Globular proteins (including type II antifreeze [11] and fast-folding proteins [12]), as well as the vast majority of individual protein domains [57], have been found to exhibit strong agreement between O and T. Such proteins have been characterized by low values of RD (T–O–R) and high values of TvO correlation coefficients. We claim that their structure is the product of cooperative (synergistic) tendencies where the residue chain—As a whole—Aligns to the external force field supplied by the aqueous solvent and, in the process of doing so, generates a prominent, centralized hydrophobic core. The Materials and Methods section contains specific formulae for calculating each of the presented values.

Results are presented in the form of FOD parameter sets. Fragments identified as consistent with the 3D Gaussian function are thought to contribute to the internalization of hydrophobic residues and exposure of hydrophilic residues on the surface. As already remarked, an analysis was performed for superfibrils, protofibrils and individual chains (with a separate 3D Gaussian function constructed for each of these structures); moreover, in the case of superfibrils and protofibrils, we also identified the status of centrally located chains, treated as parts of the larger structures’ undergoing analysis. The identification of residues which locally oppose the 3D Gaussian distribution followed the procedure outlined in the Materials and Methods section.

### 2.2. Comparative Analysis of Individual Chains

The status of individual chains was expressed by RD and correlation coefficients. When computing these values, chains may have been treated as standalone structural units or as parts of larger structures, such as proto- and super-fibrils (with a suitable 3D Gaussian function that encapsulates the entire structure in each case). The values listed in Table 1 indicate that selected chains adopted fibrillar conformations: This was evidenced by high values of RD and correlation coefficients biased towards intrinsic hydrophobicity distributions (in some cases, HvT and TvO dipped into the negative territory). These properties were commonly observed, regardless of whether the chain was analyzed on its own or as part of a larger fibril. This suggests that the fibril’s overall structural pattern forced individual chains to adopt conformations which prevented proper alignment with the aqueous solvent. The resulting need for the internalization of hydrophobic residues caused chains to aggregate and form larger fibrils in which hydrophobic residues were tightly clustered and shielded from water.

A comparative analysis showed that the tau protein was the most prominent example of an amyloid structure—At least under the FOD criteria.

### 2.3. Protofibril Structure

The characterization of the distributions of hydrophobicity within protofibrils using the FOD model (Table 2) revealed substantial diversity. In this case, tau amyloids were regarded as the best examples of a purely amyloid-like structure. This was evidenced by their negative HvT and TvO correlation coefficients, strongly positive values of the HvO coefficient, and high values of RD.

While RD remained high for all presented amyloid forms, indicating strong deviations from the monocentric core pattern, the correlation coefficients themselves varied in value (and were only negative in the case of the tau amyloid). Relatively speaking, the smallest deviations from the globular structure were observed for the ASyn amyloid.

### 2.4. Superfibril Structure

The characteristics of superfibrils, listed in Table 3, revealed disagreement between O and T. Comparing all presented parameters suggested that these structures were far removed from globular forms (as indicated by high values of RD—Although these values were lower than for all protofibrils except the tau amyloid). The variability of correlation coefficients was also lower than in the case of protofibrils, although for the tau amyloid, this variability appeared to be biased towards HvO. This observation suggested that the superfibril may have emerged as a way to limit deviations from the entropically favorable distribution of hydrophobicity (expressed by T).

**Figure 1:**
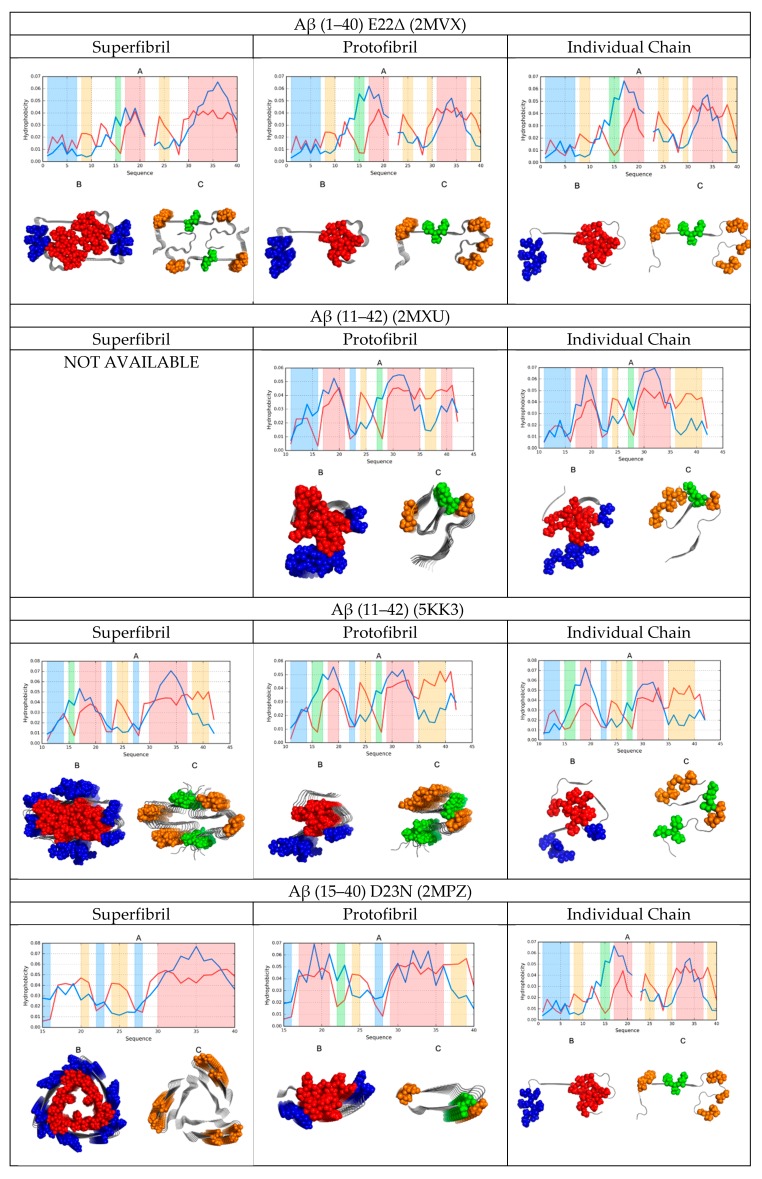

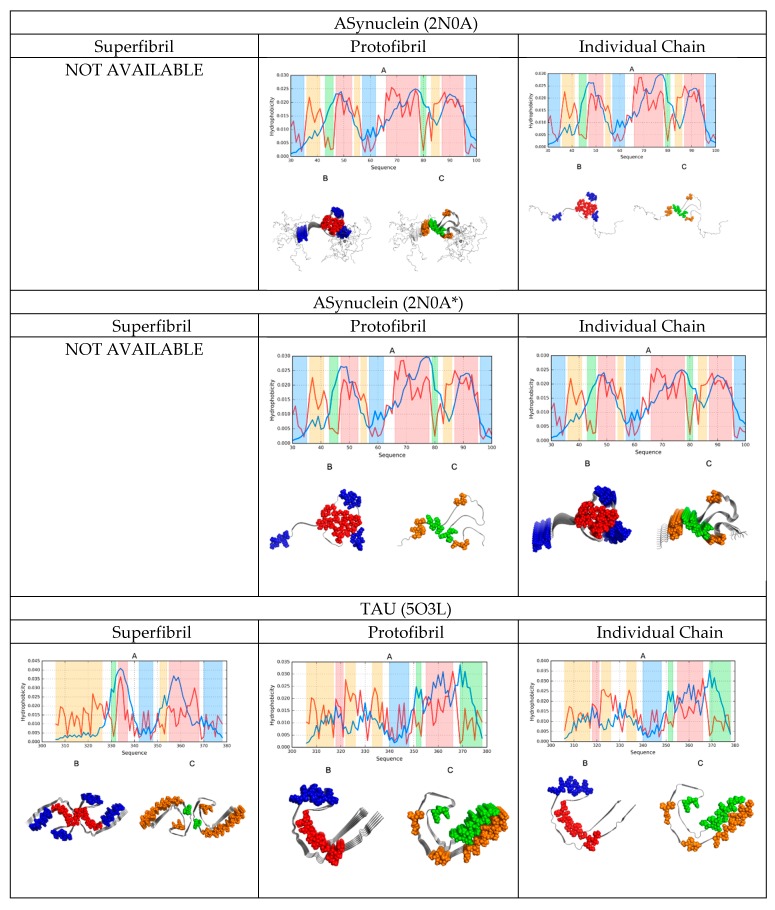
Distribution of hydrophobicity in superfibrils, protofibrils, and individual chains of the presented amyloids. A 3D visualization reveals the placement of accordant fragments (hydrophobic core—Red; hydrophilic surface—Blue), as well as fragments that deviate from the theoretical model (hydrophobicity exposed on the surface—Orange; internalized hydrophilic residues—Green). The same color coding was applied in the attached profiles.

A graphical depiction of all discussed structures is provided in Figure 1.

### 2.5. Status of the Inter-Fibril Interface

The fuzzy oil drop model posits that protein complexes are produced by exposing hydrophobic residues on the protein surface where they may attract hydrophobic residues exposed by other proteins. The interface itself, if located in the interior of a protein complex, may contribute to a complex-wide hydrophobic core. Regardless of the FOD status of the complex as a whole, if the hydrophobicity of the interface is locally consistent with the theoretical (Gaussian) distribution plotted for the complex, that interface is regarded as having a stabilizing influence on the resulting structure.

**Figure 2:**
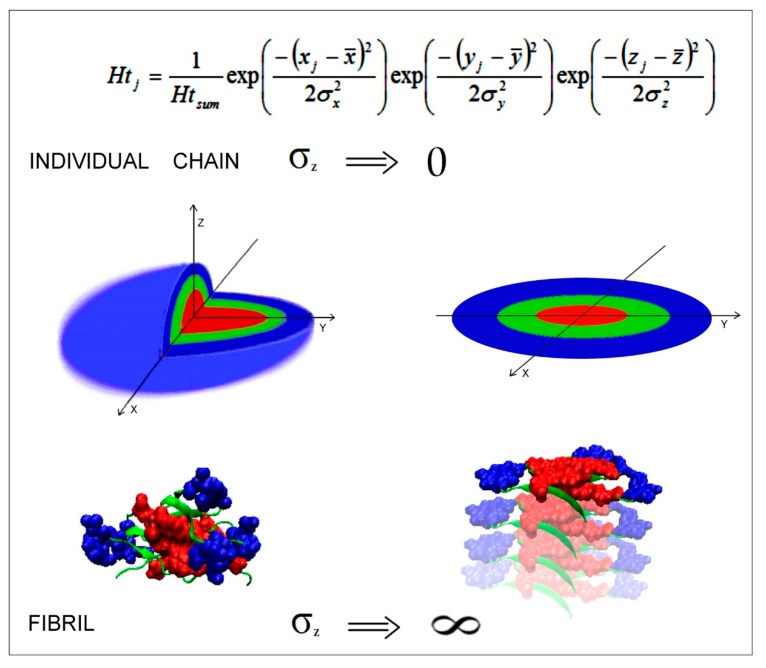
3D Gaussian distribution of hydrophobicity in a globular protein immunoglobulin (IgG light chain, V domain)—Left—And 2D Gaussian distribution of hydrophobicity in its amyloid counterpart (PDB ID = 6HUD [58])—Right. The transformation of the final term of the Gaussian function explains the properties of individual fibrils (top) and ribbonlike micelles (bottom). Red fragments exhibited strong hydrophobicity density in the center of the molecule, green fragments exhibited moderate hydrophobicity, and blue fragments exhibited a low hydrophobicity on the surface. The V domain was taken as an example of a globular form that was close enough to be accordant with the FOD model (see details in [59]).

Table 4 lists the status of interface fragments in selected amyloids (consisting of two or three protofibrils each), which suggested that the aqueous solvent plays an important role in complexation. The presence of a hydrophobic core, as shown in Figure 2, also pointed to the conclusion that a ribbon-like micelle may be constructed in various ways, depending on the properties of individual protofibrils.

The superfibril formation model, proposed on the basis of the presented research, asserts that the superfibril emerges as a result of interactions with the environment where each protofibril exposes a complexation interface. The interface is subsequently shielded from contact with water by contacting another protofibril (as evidenced by its RD value, which remains below 0.5). As a result, protofibrils cluster together to prevent hydrophobicity from being exposed to the solvent.

An exception to the above rule was observed in the case of tau amyloid as appeared in tau amyloid as appeared in 5O3T. Here, the dominant influence of intrinsic hydrophobicity upon the structure of the interface suggested a different superfibril formation mechanism.

The status of the Aβ (1–42) as appeared in 5KK3 interface suggested a deviation from the fuzzy oil drop model. This was due to the strongly micellar characteristics of individual protofibrils (amyloid PDB ID 2MXU). Individual chains (when analyzed as part of the protofibril) slightly exceeded the RD = 0.5 threshold.

### 2.6. Comparative Analysis

A comparative analysis of the status of superfibrils, protofibrils, and individual chains in amyloids, as shown in Figure 1 in the form of color-coded 3D visualizations and profiles comparing O with T, revealed the presence of a hydrophobic core in fibrillar structures. Cross-sections revealed that this core was centrally located. However, it should be noted that in this case, the core adopted the form of a linear band, engaging parts of each protofibril—As revealed by the successive horizontal slices of the resulting superfibril. Each protofibril contributed to this core in a similar manner, although some small differences could be observed. More significant differences were revealed when analyzing the hydrophobic core of each chain in separation: here, many chains lacked any concentration of hydrophobicity.

Comparing theoretical and observed distributions of hydrophobicity revealed fragments which did not conform to the theoretical model, as well as those which participated in forming a shared hydrophobic core. In all presented amyloids, hydrophobic residues tended to congregate in the central part of the proto- or super-fibril, although the effect was less pronounced in the case of the tau amyloid (particularly in the structure of 5O3T).

The values calculated for proto- and super-fibrils enabled us to speculate about their potential formation mechanisms. Aβ (1–42) structures exhibited a greater discordance as protofibrils than as superfibrils (as revealed by their corresponding correlation coefficients and RD values), whereas the opposite was true for the tau amyloid: here, the protofibril was more consistent with the theoretical model than the corresponding superfibril (except for the 5O3T form).

In all cases, α-synuclein was revealed as particularly consistent with the monocentric core model. This was confirmed by analyzing its cross-section, where the long fragment outlying the fibril exhibited good accordance with the theoretical distribution. This fragment shielded the core and ensured entropically favorable contact with water by exposing multiple polar residues (Figure 1).

In conclusion, we could identify a certain synergy between individual chains that assembled to produce a concentric distribution of hydrophobicity visible in the cross-section of the fibril. This phenomenon was also evident when comparing the status of individual chains—They exhibited better alignment with the theoretical model when analyzed as part of the amyloid fibril than as standalone structures.

The obtained data also provided clues to the formation of superfibrils, including why, in some cases, such structures failed to emerge. An interesting case was the Aβ (11–42) amyloid, which exists both as a fibril (2MXU) and as a superfibril (5KK3). Comparing the FOD parameters for both forms suggested that the protofibrillar form was preferred. This was further confirmed by the misalignment between the observed and theoretical hydrophobicity distributions in the interface zone of 5KK3.

High values of RD (Table 1, Table 2, Table 3 and Table 4) emerged as a result of the linear arrangement of alternating bands of high and low hydrophobicity, which did not conform to the monocentric core pattern. However, when we eliminated the fragments responsible for this distribution, the remaining fragments were found to conform to the theoretical model by internalizing hydrophobic residues and assuming a quasi-micellar form. This was evident in nearly all super- and proto-fibrillar structures illustrated in Figure 1.

### 2.7. Mathematical Differentiation of Globular Proteins and Amyloids

A structural analysis of all amyloids listed in the PDB revealed wholly planar structures that were otherwise unobserved in biologically-active proteins. From the point of view of the fuzzy oil drop model, the distribution of hydrophobicity in amyloid fibrils may be modeled by a 2D rather than a 3D Gaussian function. Hydrophobic residues tend to congregate in the central part of the ribbonlike micelle, as predicted by a 2D Gaussian distribution. Figure 2 illustrates the differences between the 3D Gaussian distribution (observed in globular proteins) and the corresponding 2D distribution (present in amyloid fibrils).

## 3. Discussion

The observed diversity of composition, length, secondary structural features and spatial arrangement of superfibrils provided a rich field upon which to generalize the amyloid transformation mechanisms. We should also keep in mind that, for example, α-synuclein has been found in complexes with presynaptic terminals in vivo or micelles in vitro [60]. As described in [61,62,63,64], the polypeptide chains discussed in this paper could not, by themselves, adopt a globular conformation (i.e., become a spherical micelle with a centralized hydrophobic core). In such cases, the optimization of interactions with the aqueous solvent was achieved by forming a ribbonlike micelle, where only the terminal chains were hydrophobically frustrated. Such frustration could, however, be resolved by attracting additional chains, which explained the rapid growth of fibrils.

The fuzzy oil drop model is applicable to a broad variety of proteins, from globular structures, highly consistent with T, to structures where local discordance can be observed (usually manifesting as a local excess of hydrophobicity associated with complexation sites or a local hydrophobicity deficiency associated with ligand binding pockets), as discussed in [61]. Examples of amyloid proteins (Aβ and tau), analyzed in the context of potential alternative conformations predicted by specialized folding simulation software, are provided in [62,63,64].

The presented analysis of amyloid superfibrils revealed the ubiquitous tendency to eliminate contact between hydrophobic surfaces and the polar solvent. This process usually resulted in the generation of globular structures which corresponded—With varying accuracy—To the spherical micelle model. When no such conformation could be obtained, the interaction between hydrophobic residues and the surrounding water instead led to the formation of ribbonlike micelles, of which amyloid fibrils are an example.

Superfibrils emerge as a result of the complexation of hydrophobic protofibril fragments which cluster together to exclude water. Similarly, protofibrils themselves often possess hydrophobic cores that are formed by residues which are not directly engaged in complexation. The greatest deviations from the monocentric core model were observed in individual chains, as revealed by their high RD values (with the tau amyloid being the sole exception). This proposed mechanism of complexation was supported by the relatively low RD values observed in interfaces, indicating that their component residues obeyed the theoretical hydrophobicity distribution model.

The in vitro amyloid transformation of globular proteins requires additional stimuli [65]. For example, shaking causes the aeration of water, which increases its phase boundary area. Under normal conditions, water provides proteins with a force field that guides the folding process, leading to the formation of a centralized core; however, when this is not possible (as suggested by folding simulations involving the presented amyloid polypeptides), a ribbonlike micelle may emerge instead.

The issue of whether certain amino acid sequences are particularly predisposed to generating amyloid structures is further discussed in [66]. Molecular dynamics simulations carried out in the course of analyzing structural rearrangements implicated in amyloid transformation have revealed that many different sequences are able to undergo such a transformation [67]. In particular, mutations that reduce solubility seem to promote the formation of fibrillar forms [68]. The AMYLPRED2 database provides a comprehensive overview of the propensity of various sequences to undergo fibrillation [69], although its accuracy remains unsatisfactory [70].

The specific FOD properties of amyloid properties, as opposed to globular proteins, were confirmed by carrying out an analysis of a large set of nonredundant proteins and individual protein domains. It has been determined that the vast majority of such structures conform to the theoretical distribution (T) [57].

## 4. Materials and Methods

### 4.1. Data

The analysis concerned a number of amyloid structures (Table 5) listed in the Protein Data Bank [18]. The presented forms differed with respect to sequence, chain length, the conformation of beta folds, protofibril structure, and the arrangement of protofibrils in each superfibril. Structural data were obtained using solid state NMR imaging [71,72].

All presented chains exhibited amyloid-like properties along their entire chain length except for ASyn, where only the 30–100 fragment formed a fibril, with the remaining fragments described as random coils.

The tau amyloid was peculiar in that it could form three distinct superfibrils depending on which parts of the chain act as the interface between individual fibrils.

In 2MXU, the Aβ (11–42) chain existed as an individual fibril, while it formed a superfibril consisting of two protofibrils in 5KK3. In all three cases, the structure of the fibril (the arrangement of beta folds and turns) was very similar.

### 4.2. The fuzzy Oil Drop Model (FOD)

As the fuzzy oil drop model applied in our analysis has already been presented in publications [16,17], we now limit ourselves to a brief recapitulation of its conceptual framework.

The model asserts that the distribution of hydrophobicity in a globular protein can be modeled using a 3D Gaussian function. The Gaussian form is constructed in such a way that the entire protein fits in an axis-aligned ellipsoid capsule whose dimensions are given (via σ coefficients) as 1/6 of the distance between the most distal atoms in each orthogonal direction (the so-called three-sigma rule). The status of each amino acid, represented by its effective atom (i.e., the averaged-out position of all atoms belonging to a given residue), is then obtained by computing the value of the Gaussian function at its exact position. This procedure yields a distribution of hydrophobicity that is referred to as the theoretical (or idealized) distribution (T).

The applied 3D Gaussian formula is as follows via Equation (1):

(1) $$\tilde{H}t_{j}=\frac{1}{\tilde{H}t_{sum}}\exp\left(\frac{-{\left(x_{j}-\bar{x}\right)}^{2}}{2\sigma_{x}^{2}}\right)\exp\left(\frac{-{\left(y_{j}-\bar{y}\right)}^{2}}{2\sigma_{y}^{2}}\right)\exp\left(\frac{-{\left(z_{j}-\bar{z}\right)}^{2}}{2\sigma_{z}^{2}}\right)$$

where $\tilde{H}t_{j}$ describes the theoretical hydrophobic density (hence the *t* index) at the position of the effective atom of *j*-th residue. The mean of this 3D Gaussian function is located at the origin.

The theoretical distribution is confronted with the distribution of observed (or empirical) hydrophobicity density (O), which expresses the hydrophobic interactions within the protein body.

In order to calculate O_j_, we applied the following formula [78], shown here as Equation (2):

(2) $$\tilde{H}o_{j}=\frac{1}{\tilde{H}o_{sum}}\sum_{i=1}^{N}(H_{i}^{r}+H_{j}^{r})\left\{ \begin{array}{l} \left[1-\frac{1}{2}\left(7{\left(\frac{r_{ij}}{c}\right)}^{2}-9{\left(\frac{r_{ij}}{c}\right)}^{4}+5{\left(\frac{r_{ij}}{c}\right)}^{6}-{\left(\frac{r_{ij}}{c}\right)}^{8}\right)\right]\;\mathrm{for}\;r_{ij}\leq c\\ 0\;\mathrm{for}\;r_{ij}>c \end{array}\right.$$

where *N* is the number of amino acids in the protein, ${\tilde{H}}_{i}^{r}$ is the hydrophobic parameter of the *i*-th residue, *r_ij_* is the distance between effective atoms of two interacting residues, and C is the hydrophobic interaction cutoff distance, assumed to be 9 Å.

In order to compare the T and O distributions, we applied the Kullback–Leibler divergence entropy formula [56], shown here as Equation (3):

(3) $$D_{KL}(p|p^{0})=\sum_{i=1}^{N}p_{i}\log_{2}(p_{i}/p_{i}^{0})$$

where $p_{i}$ is the target probability, $p_{i}^{0}$ is the reference probability, *N* is the number of samples in the dataset (here being the number of residues). In an FOD model, the target is O, and the references are T (Equation (1)) and R (“random”). In the latter case, each residue is assigned a value of 1/N, yielding a uniform distribution of hydrophobicity—The “opposite” to a 3D Gaussian distribution. The status of O is then obtained by comparing the value of *D_KL_*(O|T) with the value of *D_KL_*(O|R). If *D_KL_*(O|T) ≥ *D_KL_*(O|R), O is assumed to approximate R more than T, indicating the lack of a prominent hydrophobic core, while the opposite, *D_KL_(O|T)* < *D_KL_*(O|R), means that a hydrophobic core is established in the given protein. Since we were interested in the relation between *D_KL_*(O|T) and *D_KL_*(O|R), to avoid having to deal with two values, we combined them into one normalized value called RD—The relative distance Equation (4):

(4) $$RD=\frac{O|T}{O|T+O|R}$$

RD < 0.5 suggests that O is “closer” to T than to R, and the structure therefore contains a hydrophobic core, a status also called “an accordance with the FOD model;” otherwise, the observed distribution is more closely aligned to the flat distribution, and no prominent core can be observed. The eq. 4. yields a value of RD for the T–O–R relationship. By replacing R with H (the distribution of intrinsic hydrophobicity, containing the normalized values of hydrophobicity parameters of residues), we obtained another value of RD that corresponded to the T–O–H relation. In this case, a value greater than 0.5 indicated that the structure was dominated by the intrinsic hydrophobicity of individual residues and lacked a shared hydrophobic core.

Going back to the differences between T and O, we could identify fragments where the two distributions were (a) highly accordant, (b) locally discordant, or (c) in opposition to each other. When dealing with a strongly discordant structure, we identified the fragments responsible for this effect by progressively eliminating discordant residues until the value of RD dropped below 0.5. The identification of residues which comprised the hydrophobic core was performed by looking for those that expressed high values of both T and O (relative to other residues in the same protein).

### 4.3. Comparative Analysis

As discussed above, each of the presented amyloids was characterized by using two RD parameters and three correlation coefficient values. The structural analysis involved superfibrils, protofibrils and individual chains. In each case, a suitable 3D Gaussian function was generated for the structure in question. In addition, when analyzing superfibrils and protofibrils, we also identified the status of their constituent chains (focusing on centrally located chains as the most representative examples of arbitrarily long fibrils). A similar analysis of the influence of water on protein structure was provided in [79], where three different types of variable domains were obtained during crystallization, depending on water conditions (pH, ionic strength).

## 5. Conclusions

In summary, by combining the above results with those presented in [16,17,80], we can claim that the aqueous solvent drives the folding process via hydrophobic interactions, leading to structures which approximate the 3D Gaussian distribution (in the case of globular proteins), whereas amyloid transformation instead results in a 2D Gaussian distribution where the third dimension (corresponding to the length of the ribbonlike micelle) tends to infinity. As a result, each cross-section of the fibril exhibits a similar distribution of hydrophobicity that peaks in its central part. Fragments which deviate from theoretical expectations pay a “price” for generating an imperfect core, but they are nevertheless capable of shielding their strongly hydrophobic residues from direct contact with water.

## Figures and Tables

**Table 1:** Summary of parameters describing the status of individual chains as both standalone units and as part of larger structures (where the 3D Gaussian function was constructed for a protofibril and for a superfibril). * Only the 30–100 fragment, which exhibited amyloid-like properties, was subjected to analysis. The detailed data are given in Supplementary Materials.

RD		Correlation Coefficient			Amyloid
T–O–R	T–O–H	HvT	TvO	HvO	
**Chain as Part of Superfibril**					
0.554	0.475	0.452	0.558	0.843	Aβ (15–40) D23N (2MPZ)
0.607	0.620	0.459	0.664	0.784	Aβ (1–40) E22Δ (2MVX)
0.565	0.594	0.395	0.466	0.782	Aβ (11–42) (5KK3)
0.747	0.697	0.012	0.265	0.696	TAU (5O3L)
0.761	0.722	0.010	0.224	0.731	TAU (5O3O)
0.728	0.666	−0.019	0.136	0.785	TAU (5O3T)
**Chain as Part of Protofibril**					
0.491	0.487	0.382	0.458	0.839	Aβ (15–40) D23N (2MPZ)
0.649	0.686	0.310	0.322	0.779	Aβ (1–40) E22Δ (2MVX)
0.513	0.620	0.404	0.471	0.849	Aβ (11–42) (2MXU)
0.569	0.600	0.299	0.286	0.789	Aβ (11–42) (5KK3)
0.664	0.595	−0.022	0.083	0.767	TAU (5O3L)
0.661	0.607	−0.012	0.089	0.772	TAU (5O3O)
0.688	0.618	−0.029	0.098	0.782	TAU (5O3T)
0.506	0.588	0.285	0.506	0.823	ASYN (2N0A *)
**Chain as Individual Unit**					
0.626	0.467	0.355	0.351	0.615	Aβ (15–40) D23N (2MPZ)
0.635	0.562	0.295	0.363	0.615	Aβ (1–40) E22Δ (2MVX)
0.536	0.519	0.408	0.567	0.697	Aβ (11–42) (2MXU)
0.660	0.555	0.355	0.263	0.698	Aβ (11–42) (5KK3)
0.674	0.410	−0.039	0.091	0.545	TAU (5O3L)
0.679	0.430	−0.027	0.095	0.548	TAU (5O3O)
0.683	0.415	−0.033	0.096	0.551	TAU (5O3T)
0.784	0.727	0.099	0.208	0.725	ASYN (2N0A *)

**Table 2:** Summary of fuzzy oil drop model (FOD) parameters for amyloid protofibrils. Underscores indicate that the given amyloid did not form a superfibril; * Only the 30–100 fragment, which exhibited amyloid-like properties, was subjected to analysis. The detailed data are given in SI.

RD		Correlation Coefficient			Amyloid
T–O–R	T–O–H	HvT	TvO	HvO	PDB ID
0.614	0.600	0.262	0.412	0.786	Aβ (15–40) D23N (2MPZ)
0.639	0.659	0.280	0.365	0.718	Aβ (1–40) E22Δ (2MVX)
0.680	0.756	0.246	0.363	0.821	Aβ (11–42) (2MXU)
0.608	0.623	0.235	0.335	0.750	Aβ (11–42) (5KK3)
0.652	0.564	−0.022	0.145	0.705	TAU (5O3L)
0.652	0.0.577	−0.012	0.151	0.712	TAU (5O3O)
0.674	0.584	−0.028	0.152	0.720	TAU (5O3T)
0.531	0.598	0.241	0.492	0.798	ASYN (2N0A *)

**Table 3:** Summary of FOD parameters for superfibrils. The detailed data are given in Supplementary Materials.

RD		Correlation Coefficient			Amyloid
T–O–R	T–O–H	HvT	TvO	HvO	
0.578	0.494	0.394	0.554	0.790	Aβ (15–40) D23N (2MPZ)
0.590	0.592	0.438	0.674	0.727	Aβ (1–40) E22Δ (2MVX)
0.620	0.652	0.330	0.440	0.756	Aβ (11–42) (5KK3)
0.730	0.662	0.014	0.297	0.643	TAU (5O3L)
0.745	0.687	0.012	0.259	0.675	TAU (5O3O)
0.724	0.641	0.008	0.301	0.716	TAU (5O3T)

**Table 4:** FOD parameters for amyloids consisting of two or three protofibrils. In the case of tau proteins as appeared in 5O3T, due to its asymmetric conformation, the number 321 and 323 residues were contributed by one protofibril, while the remaining residues belonged to its partner. Underscores indicate that the interface residues formed a beta fold. The detailed data are given in Supplementary Materials.

RD		Correlation Coefficient			Amyloid	
T–O–R	T–O–H	HvT	TvO	HvO	Amyloid	Residues in Interface
0.424	0.155	0.452	0.717	0.768	Aβ (15–40) D23N	17, 28, 29, 31, 38, 40
0.454	0.313	0.375	0.709	0.623	Aβ (1–40) E22Δ	3, 4, 13, 28–30, 37–40
0.595	0.707	0.35	0.425	0.591	Aβ (11–42))	11, 13, 15, 17, 34–38
0.388	0.532	0.612	0.828	0.811	TAU (5O3L)	331–336, 338
0.401	0.550	0.666	0.754	0.950	TAU (5O3O)	331–336
0.538	0.314	−0.189	−0.069	0.809	TAU (5O3T)	321, 323/313, 15, 317

**Table 5:** Summary of amyloids subjected to analysis, along with the brief characteristics of each amyloid. * Only the 30–100 fragment in a 140aa chain exhibited amyloid-like properties. NCP: number of chains in a protofibril; NPF: number of protofibrils in a superfibril. α-synuclein is also referred to as “ASyn.”.

Name	PDB ID	Chain Length	Fragment	Mutation	Beta-Structure (%)	NCPxNPF	Ref.
Aβ (15**–**40)	2MPZ	26 aa	15**–**40	D23N	56	9 × 3	[73]
Aβ (11**–**42)	2MXU	32 aa	11**–**42		68	12 × 1	[74]
Aβ (1**–**40)	2MVX	39aa	1**–**40	E22Δ	35	5 × 2	[73]
Aβ (11**–**42)	5KK3	32 aa	11**–**42		59	9 × 2	[75]
TAU	5O3L	73 aa	306**–**378		13	5 × 2	[76]
	5O3O	73 aa	306**–**378		13	5 × 2	[77]
	5O3T	73 aa	306**–**378		13	5 × 2	[78]
ASyn	2N0A	140/70 aa*	1**–**140 *		92	10 × 1	[79]

## Supplementary Materials

The following are available online at.

## Abbreviations

α-synuclein	Asyn
