# Supplementary material for: The Amyloid as a Ribbon-Like Micelle in Contrast to Spherical Micelles Represented by Globular Proteins
Source: Molecules. 2019 Dec 3;24(23):4395. doi: 10.3390/molecules24234395 (PMC6930452; doi:10.3390/molecules24234395)
Supplement: Supplementary file 1 [file molecules-24-04395-s001.pdf]

molecules

Table 1S. Summary of parameters describing the status of individual chains – as standalone units and as part of larger structures (where the 3D Gaussian is constructed for a protofibril and for a superfibril respectively). * – only the 30-100 fragment, which exhibits amyloid-like properties, is subjected to analysis. Underscores indicate that the given amyloid does not form a superfibril; D_{KL} values for T, R and H distribution treated as target distribution for O distribution allowing the RD calculation.

RD		D_{KL}			AMYLOID
T-O-R	T-O-H	O|T	O|R	O|H	
**CHAIN AS PART OF SUPERFIBRIL**					
0.554	0.475	0.163	0.131	0.180	Aβ(15-40) D23N (2MPZ)
0.607	0.620	0.242	0.156	0.148	Aβ (1-40) E22Δ (2MVX)
0.565	0.594	0.218	0.168	0.149	Aβ (11-42) (5KK3)
0.747	0.697	0.701	0.236	0.304	TAU (5O3L)
0.761	0.722	0.738	0.231	0.284	TAU (5O3O)
0.728	0.666	0.576	0.214	0.288	TAU (5O3T)
**CHAIN AS PART OF PROTOFIBRIL**					
0.491	0.487	0.135	0.140	0.142	Aβ(15-40) D23N (2MPZ)
0.649	0.686	0.315	0.170	0.144	Aβ (1-40) E22Δ (2MVX)
0.513	0.620	0.175	0.166	0.107	Aβ (11-42) (2MXU)
0.569	0.600	0.230	0.174	0.153	Aβ (11-42) (5KK3)
0.664	0.595	0.422	0.213	0.287	TAU (5O3L)
0.661	0.607	0.424	0.217	0.274	TAU (5O3O)
0.688	0.618	0.471	0.213	0.291	TAU (5O3T)
0.506	0.588	0.254	0.247	0.178	ASYN (2N0A*)
**CHAIN AS INDIVIDUAL UNIT**					
0.626	0.467	0.241	0.143	0.275	Aβ(15-40) D23N (2MPZ)
0.635	0.562	0.345	0.197	0.268	Aβ (1-40) E22Δ (2MVX)
0.536	0.519	0.204	0.176	0.189	Aβ (11-42) (2MXU)
0.660	0.555	0.289	0.148	0.231	Aβ (11-42) (5KK3)
0.674	0.410	0.371	0.179	0.532	TAU (5O3L)
0.679	0.430	0.388	0.183	0.512	TAU (5O3O)
0.683	0.415	0.393	0.182	0.552	TAU (5O3T)
0.784	0.727	0.889	0.244	0.333	ASYN (2N0A*)

Table 2S. Summary of FOD parameters for amyloid protofibrils. Underscores indicate that the given amyloid does not form a superfibril; * – only the 30-100 fragment, which exhibits amyloid-like properties, is subjected to analysis. D_{KL} values for T, R and H distribution treated as target distribution for O distribution allowing the RD calculation.

RD		D_{KL}			AMYLOID
T-O-R	T-O-H	O|T	O|R	O|H	PDB ID
0.614	0.600	0.241	0.151	0.160	Aβ(15-40) D23N (2MPZ)
0.639	0.659	0.335	0.188	0.173	Aβ (1-40) E22Δ (2MVX)
0.680	0.756	0.369	0.173	0.118	<u>Aβ (11-42) (2MXU)</u>
0.608	0.623	0.277	0.178	0.168	Aβ (11-42) (5KK3)
0.652	0.564	0.425	0.227	0.329	TAU (5O3L)
0.652	0.577	0.430	0.229	0.315	TAU (5O3O)
0.674	0.584	0.469	0.226	0.333	TAU (5O3T)
0.531	0.598	0.286	0.252	0.192	<u>ASYN (2N0A*)</u>

Table 3S. Summary of FOD parameters for superfibrils. D_{KL} values for T, R and H distribution treated as target distribution for O distribution allowing the RD calculation.

RD		D_{KL}			AMYLOID
T-O-R	T-O-H	O|T	O|R	O|H	
0.578	0.494	0.194	0.141	0.199	Aβ(15-40) D23N (2MPZ)
0.590	0.592	0.252	0.174	0.173	Aβ (1-40) E22Δ (2MVX)
0.620	0.652	0.280	0.171	0.149	Aβ (11-42) (5KK3)
0.730	0.662	0.677	0.250	0.345	TAU (5O3L)
0.745	0.687	0.712	0.243	0.323	TAU (5O3O)
0.724	0.641	0.599	0.228	0.335	TAU (5O3T)

Table 4S. FOD parameters for amyloids consisting of two or three protofibrils. In the case of 5O3T, due to its asymmetric conformation, residues no. 321 and 323 are contributed by one protofibril, while the remaining residues belong to its partner. Underscores indicate that the interface residues form a beta fold. D_{KL} values for T, R and H distribution treated as target distribution for O distribution allowing the RD calculation.

**RD**		D_{KL}			**AMYLOID**	
**T-O-R**	**T-O-H**	**O|T**	**O|R**	**O|H**	**AMYLOID**	**Residues in interface**
0.424	0.155	0.073	0.100	0.400	Aβ(15-40) D23N	17,28,29,31,38,40
0.454	0.313	0.117	0.141	0.257	Aβ (1-40) E22Δ	3,4,13,28-30,37-40
0.595	0.707	0.243	0.165	0.100	Aβ (11-42))	11, 13, 15, 17, 34-38
0.388	0.532	0.188	0.297	0.165	TAU (5O3L)	331-336, 338
0.401	0.550	0.345	0.514	0.282	TAU (5O3O)	331-336
0.538	0.314	0.357	0.307	0.779	TAU (5O3T)	321, 323 / 313, 15,317
